# Evaluation of serological assays for the diagnosis of childhood tuberculosis disease: a study protocol

**DOI:** 10.1186/s12879-024-09359-0

**Published:** 2024-05-10

**Authors:** Daniela Neudecker, Nora Fritschi, Thomas Sutter, Lenette L Lu, Pei Lu, Marc Tebruegge, Begoña Santiago-Garcia, Nicole Ritz

**Affiliations:** 1 Mycobacterial and Migrant Health Research Group, Department of Clinical Research, University of Basel Children’s Hospital Basel, University of Basel, Spitalstrasse 33, Basel, CH-4031 Switzerland; 2 University of Basel Children’s Hospital Basel, University of Basel, Basel, Switzerland; 3 Department of Computer Science, Medical Data Science, Eidgenössische Technische Hochschule (ETH) Zurich, Zurich, Switzerland; 4 Department of Immunology, UT Southwestern Medical Center, Dallas, TX USA; 5 Parkland Health and Hospital System, Dallas, TX USA; 6 Division of Geographic Medicine and Infectious Diseases, Department of Internal Medicine, UT Southwestern Medical Center, Dallas, TX USA; 7 Department of Paediatrics, The Royal Children’s Hospital Melbourne, The University of Melbourne, Parkville, Australia; 8 Department of Infection, Immunity and Inflammation, UCL Great Ormond Street Institute of Child Health, University College London, London, UK; 9 Department of Paediatrics & National Reference Centre for Paediatric TB, Klinik Ottakring, Vienna Healthcare Group, Vienna, Austria; 10 Pediatric Infectious Diseases Department, Gregorio Marañón University Hospital, Madrid, Spain; 11 Gregorio Marañón Research Health Institute (IiSGM), Madrid, Spain; 12 Centro de Investigación Biomédica en Red de Enfermedades Infecciosas (CIBER INFEC), Instituto de Salud Carlos III, Madrid, Spain; 13 Translational Research Network in Pediatric Infectious Diseases (RITIP), Madrid, Spain; 14 Paediatric Infectious Diseases Unit, Children’s Hospital, Lucerne Cantonal Hospital, Lucerne, Switzerland

**Keywords:** Serodiagnosis, Antigen, Antibody, Paediatric, Serology, Childhood TB

## Abstract

**Background:**

Tuberculosis (TB) poses a major public health challenge, particularly in children. A substantial proportion of children with TB disease remain undetected and unconfirmed. Therefore, there is an urgent need for a highly sensitive point-of-care test. This study aims to assess the performance of serological assays based on various antigen targets and antibody properties in distinguishing children (0–18 years) with TB disease (1) from healthy TB-exposed children, (2) children with non-TB lower respiratory tract infections, and (3) from children with TB infection.

**Methods:**

The study will use biobanked plasma samples collected from three prospective multicentric diagnostic observational studies: the Childhood TB in Switzerland (CITRUS) study, the Pediatric TB Research Network in Spain (pTBred), and the Procalcitonin guidance to reduce antibiotic treatment of lower respiratory tract infections in children and adolescents (ProPAED) study. Included are children diagnosed with TB disease or infection, healthy TB-exposed children, and sick children with non-TB lower respiratory tract infection. Serological multiplex assays will be performed to identify *M. tuberculosis* antigen-specific antibody features, including isotypes, subclasses, Fc receptor (FcR) binding, and IgG glycosylation.

**Discussion:**

The findings from this study will help to design serological assays for diagnosing TB disease in children. Importantly, those assays could easily be developed as low-cost point-of-care tests, thereby offering a potential solution for resource-constrained settings.

**ClinicalTrials.gov Identifier:**

NCT03044509.

**Supplementary Information:**

The online version contains supplementary material available at 10.1186/s12879-024-09359-0.

## Background

Diagnosing tuberculosis (TB) in children presents several challenges [1]. TB disease in children is confirmed only in about 50% of patients due to the paucibacillary nature [2, 3]. In the absence of a reliable and easily accessible diagnostic test for screening and confirming TB disease in children, diagnosis typically relies on clinical findings, TB contact history, chest radiography findings, and the results of immune-based TB tests, the Tuberculin skin test (TST) and interferon-γ release assays (IGRA) [4]. However, both immunodiagnostic tests have suboptimal performance and are not well-suited for screening for TB disease [5, 6].

Serological assays have the potential to serve as a screening tool for TB infection and disease in children, especially in resource-limited settings where advanced diagnostic methods are limited. This potential stems from their blood-based nature, thus not requiring sputum collection, and their feasibility to be used as point-of-care tests [7]. However, currently available commercial serological assays are not recommended for clinical use due to their insufficient and variable diagnostic performance, characterised by limited sensitivity, specificity, and susceptibility to cross-reactivity [8, 9]. In a recent narrative review focusing on the diagnostic performance of non-commercial serological assays for TB in children, we found that studies which measured antibodies against only one antigen generally reported relatively high specificity but only achieved limited sensitivity [10]. Higher sensitivity can be achieved when antibodies against multiple targets are measured, and results are interpreted in combination. In addition, emerging evidence suggests that certain antibody properties, such as antibody Fc receptor (FcR) binding profiles [11, 12] and antibody glycosylation patterns [13], can potentially be used to differentiate between TB infection and disease. However, most of those studies have been done in adults, and the evidence in children remains extremely limited.

## Methods

### Aim

The aim of this study is to evaluate the diagnostic performance of serological assays in detecting children with TB disease, and in distinguishing those subjects from (1) healthy TB-exposed children, (2) children with non-TB lower respiratory tract infection, and (3) children with TB infection.

### Study setting and population

This study will utilise plasma samples obtained from three different prospective multicentric observational studies: the Childhood Tuberculosis in Switzerland (CITRUS) study (NCT03044509), the Pediatric TB Research Network in Spain (pTBred), and the Procalcitonin guidance to reduce antibiotic treatment of lower respiratory tract infections in children and adolescents (ProPAED) study (ISRCTN 17,057,980) (Table 1).

**Table 1:** Overview of studies from which samples will be used in the described project, including inclusion- and exclusion criteria

	Inclusion criteria	Exclusion criteria	Study design	Time	Country
CITRUS	< 18 years Undergoing evaluation for TB exposure, TB infection, TB disease	- Anti-mycobacterial treatment more than 5 days prior to inclusion - Previous treatment for TB disease or infection	Multi-centric study with nine centres in Switzerland	Since May 2017 (on going)	Switzerland
pTBred	< 18 years Undergoing evaluation for TB exposure, TB infection, TB disease	- Anti-mycobacterial treatment more than 5 days prior to inclusion - Previous treatment for TB disease or infection	Multidisciplinary collaborative network	Oct 2019 - Jun 2021	Spain
ProPAED	pulmonary non-TB disease: < 18 years, presentation with lower respiratory tract infection including fever and cough, regardless of previous antibiotic treatment history	- Severe immunosuppression - Known HIV infection - Immunosuppressive treatment - Neutropenia - *M. tuberculosis* infection - Cystic fibrosis - Viral laryngotracheitis - Hospital stay within the previous 14 days - Other severe infections (e.g., osteomyelitis, endocarditis, or deep tissue abscesses)	Multi-centric study with two emergency departments	Jan 2009 - Feb 2010	Switzerland

CITRUS is a multicentric prospective diagnostic study done at nine centres across Switzerland (Bern, Basel, Zurich, Lausanne, Geneva, Aarau, St. Gallen, Lucerne, Bellinzona). Its primary objective is to evaluate and validate novel immunodiagnostic assays for childhood TB [14, 15]. The study includes children under the age of 18 years, with or without a history of Bacillus Calmette-Guérin (BCG) vaccination, who are undergoing evaluation for TB disease, infection, and exposure. Children who have received any anti-mycobacterial treatment for five days or more before inclusion or who have been previously treated for TB disease or infection are excluded. Recruitment for the CITRUS study began in May 2017 and is currently ongoing.

PTBred is a multidisciplinary collaborative network established in 2014 in Spain, recruiting children < 18 years with TB. Since 2017, different types of samples have been stored in the Biobank of the Gregorio Marañon Hospital or in the individual collection registered as C.0006631 in the National Biobank Collections Registry. For this study, a common protocol for sample processing was implemented in October 2019, including children with children with TB disease, infection, and exposure irrespective of their BCG-vaccination status. The pTBred and CITRUS study follow the same inclusion and exclusion criteria [16].

The ProPAED study collected samples from children and adolescents presenting with fever and cough at two emergency departments in Switzerland (Basel and Aarau), from January 2009 to February 2010. For the ProPAED study, children with severe immunocompromise or known HIV infection, those undergoing immunosuppressive treatment, children with *M. tuberculosis* infection, neutropenia, cystic fibrosis, viral laryngotracheitis, hospital stay within the preceding 14 days, or other severe infections (e.g., osteomyelitis, endocarditis, or deep tissue abscesses) were excluded [17].

### Case definitions

In this study, we will use the published criteria of compound TB case definitions proposed by Graham et al. [18]. Briefly, confirmed TB disease is defined as the presence of bacteriologically confirmed TB disease through culture or nucleic acid amplification tests (NAAT). Unconfirmed TB disease is defined as the absence of bacteriological confirmation in the presence of at least two of the following criteria: symptoms or signs suggestive of TB disease, chest radiograph consistent with TB disease, close TB exposure or immunologic evidence of *M. tuberculosis* infection, positive response to TB treatment. TB infection is defined as the presence of immunologic evidence of *M. tuberculosis* infection, including a positive TST of ≥ 5 mm (in accordance with the Swiss and Spanish guidelines [19, 20]) or a positive IGRA without meeting the criteria for confirmed or unconfirmed TB disease. Healthy TB-exposed children are defined as asymptomatic individuals with negative results on IGRA or TST test (single or repeat testing according to age, time since exposure as defined by national guidelines), making them unlikely to have TB. Children with non-TB lower respiratory tract infection will be the sick control group and are defined as presenting with fever (core body temperature ≥ 38.0° C) and at least one symptom (cough, sputum production, pleuritic pain, poor feeding) and at least one sign (tachypnea, dyspnoea, wheezing, late inspiratory crackles, bronchial breathing, pleural rub) lasting for fewer than 14 days.

### Age stratification

The study will analyse antibody concentrations and properties in children stratified into distinct age groups: 0 to < 2, 2 to < 5, 5 to < 10, and ≥ 10 years, as proposed by Cuevas et al. [21]. This stratification is crucial due to the differences and dynamics of the nature of TB disease across age. In the youngest age range (infants and children < 2 years old), disseminated diseases and heightened susceptibility to progression from TB infection to TB disease is well-documented [22]. The risks for progression from infection to disease, as well as the subsequent mortality risk following development of disease, consistently declines during childhood, reaching its lowest point between 5 and 10 years of age [23]. Transitioning into adolescence and the onset of puberty, typically beyond the age of 10 years, the phenotype of TB disease becomes more adult-like. Pulmonary TB becomes more prevalent during this phase, contributing to an upsurge in TB-related mortality rates [24, 25].

### Selected antigen targets and antibody properties for serological assay

Some previous studies in children have demonstrated improved specificities achieved by combining both protein and glycolipid antigens within serological assays [26–29]. Furthermore, several studies have illuminated the potential for heightened sensitivity through the combined analysis of multiple antigen targets, effectively overcoming the interindividual heterogeneity of the human humoral immune response to *M. tuberculosis* [26–33].

We will analyse antibodies concentrations and properties against single protein antigens, single glycolipid antigens [12, 34–40], as well as multiple antigens in combination (Table 2). The types of antigens include cell wall fractions, whole cell lysates, and total lipids of *M. tuberculosis*. The selection of protein antigens is based on results from large protein microarray studies in adults [41–46], one large multiplex bead-based study in children [31], and published and unpublished data from an adult study performed in the U.K (MIMIC study; personal communication M. Tebruegge) [47]. In order to enhance specificity, the overlap of the antigen targets for *M. tuberculosis* with Bacillus Calmette-Guérin (BCG) and other non-tuberculous mycobacteria will be reduced.

**Table 2:** Key protein, glycolipid, and multiple antigens

Type	Name	Rv number/Full name
Protein	**FbpC** (Ag85C)	Rv0129c
	**PstS3**	Rv0928
	**PstS1**	Rv0934
	**PapA4**	Rv1528
	**GarA**	Rv1827
	**Apa** (Mpt32)	Rv1860
	**FbpB** (Ag85B)	Rv1886c
	**Mpt63**	Rv1926c
	**Mpt64**	Rv1980c
	**HspX** (Acr)	Rv2031c
	**Acg**	Rv2032
	**Rv2034**	Rv2034
	**Hrp1**	Rv2626c
	**EsxO-EsxP**	Rv2346-Rv2347
	**EspA**	Rv3616c
	**FbpD** (Mpt51)	Rv3803c
	**FbpA** (Ag85A)	Rv3804c
	**EsxB** (CFP-10)	Rv3874
	**EsxA** (ESAT-6)	Rv3875
	**EsxA-EsxB** (ESAT6-CFP10)	Rv3875-Rv3874
	**EspD-EspC**	Rv3614-Rv3615
	**EspB**	RV3881c
	**Ag85 complex**	Rv3804c-Rv1886c-Rv0129c
Glycolipid	**LAM**	Lipoarabinomannan
	**PDIM**	Phthiocerol dimycocerosates
	**TDM**	Trehalose dimycolates
	**TMM**	Trehalose monomycolates
	**PGL**	Phenolic glycolipid
Multiple antigens (H37Rv)	**Cell wall fractions**	contains proteins and non-protein compounds such as mAGP of *M. tuberculosis*
	**Cell membrane fractions**	contains the cytoplasmic membrane and components of the outer lipid layer.
	**Whole cell lysates**	contains proteins, lipids and carbohydrates present within the *M. tuberculosis* bacterial cell
	**Total hypoxic lipids**	containing hypoxic culture *M. tuberculosis*
	**Total normoxic lipids**	containing normoxic culture *M. tuberculosis*

Abbreviations: Acg - alpha-crystallin homolog -coregulated gene; Acr - alpha-crystallin homolog; Ag85 complex - antigen 85 complex; Apa - alanine and proline rich secreted protein; CFP-10 - culture filtrate protein-10; ESAT-6 - early secreted antigenic target-6; EspA - ESX-1 secretion-associated protein A; EsxA/B/C/D/O/P - early secretory antigenic target homolog A/B/C/D/O/P; FbpA/B/C/D - fibronectin binding protein A/B/C/D; GarA - Glycogen accumulation regulator A; Hrp1 - hypoxic response protein 1; HspX - heat shock protein-X; Mpt32/51/63/64 - Proteins purified from Mycobacterium tuberculosis 32/51/63/64; PapA4 - polyketide synthase (PKS) associated protein; PstS1/3 - periplasmic phosphate-binding lipoprotein S1/3

**Fig. 1:**
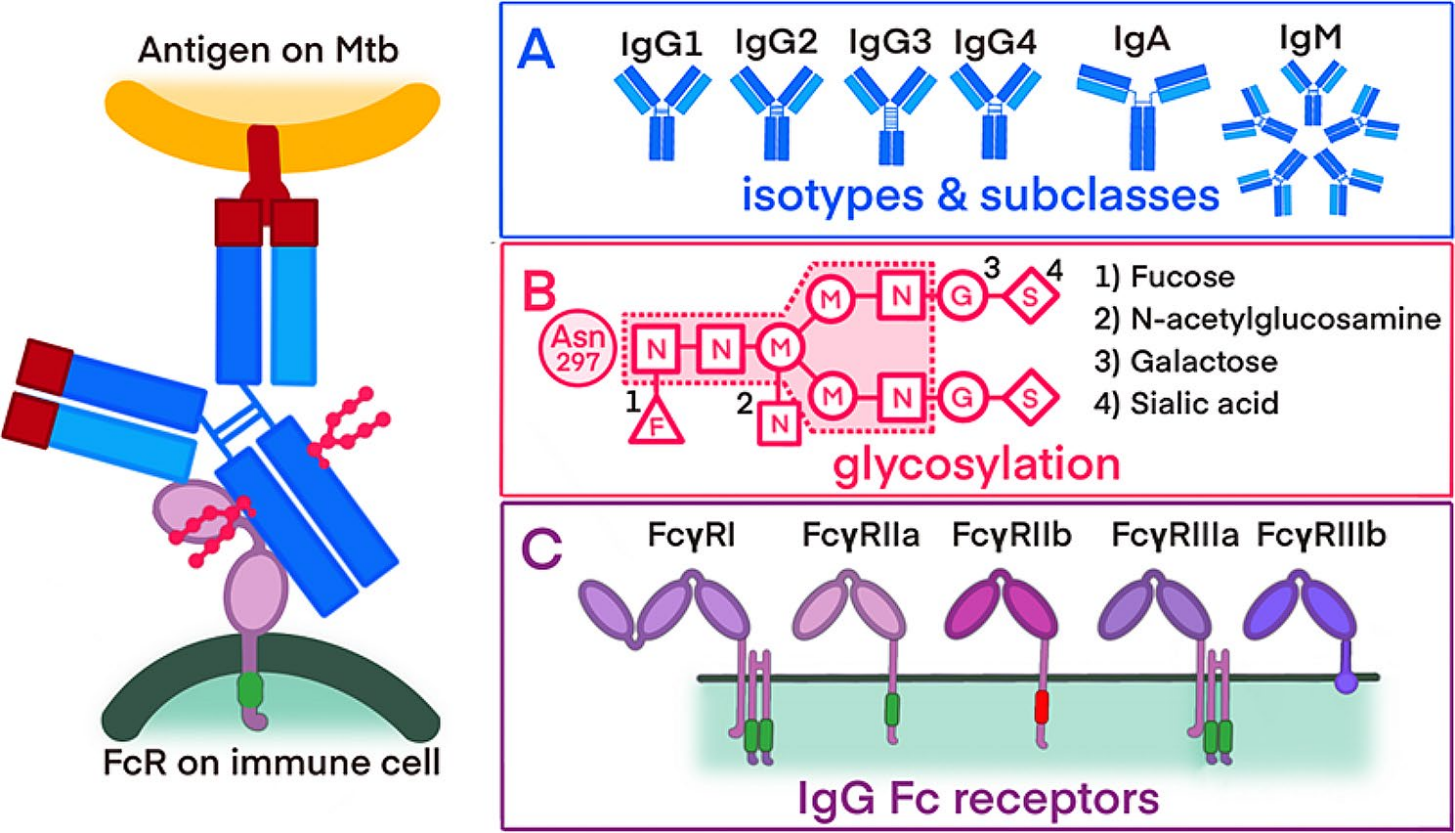
Overview of the antibody properties Interaction between the surface of *M. tuberculosis*, binding of the antibody and the recognition of the antibody by an immune cell. Sections **A**, **B**, and **C** detail the different antibody properties: **A**) antibody isotypes and IgG subclasses **B**) glycosylation patterns of antibodies, including a core glycan and potential additional sugar residues (1–4) **C**) activating and inhibiting FcRs with varying affinities for antibody binding Abbreviations: Mtb -Mycobacterium tuberculosis; FcR -fragmented crystallizable region (Fc) receptor; IgM - immunoglobulin M; IgD - immunoglobulin D, IgG_1 − 4_ - immunoglobulin G_1 − 4_; IgA - immunoglobulin A, N - N-acetylglucosamine; M - mannose; G - galactose; S - sialic acid; F - fucose

Together with targeted *M. tuberculosis* antigens, this study will evaluate the following distinct properties of the antibodies: isotypes and their subclasses, FcR binding profiles, and antibody glycosylation patterns (refer to Fig. 1). The rational for this is to obtain further information about the immune response to the antigen. TB disease results from a combination of the mycobacteria infecting and the resulting pathologic immune response. Therefore, antibody concentrations may only reflect on exposure, timepoint, and burden of mycobacteria, whereas additional properties such as FcR may reflect on the fact if the immune response producing tissue damage and pathology or not. This is shown in studies in children with TB disease that have demonstrated the potential enhancement of serological assay sensitivity through the integration of diverse antibody isotypes [48–50]. Recent advancements in adult research have indicated that an evaluation of certain antibody properties, such as FcRs binding profiles and glycosylation patterns, could potentially enable the differentiation between TB disease and infection [12, 13].

As a quality control and potential normalisation variable, we will measure the total antibody concentration of each isotype and the total antibody concentration binding to distinct FcRs.

### Sample preparation

Upon plasma sample collection, preservation is ensured through storage in a − 80 °C freezer until the initiation of laboratory assays. Customised multiplex antigen-coupled beads will be produced to evaluate antigen-specific antibodies concentrations and properties in plasma samples. The protein antigens will be coupled to carboxylated beads through covalent NHS-ester linkages, using 1-ethyl-3-(3-dimethylaminopropyl) carbodiimide hydrochloride and Sulfo-NHS (Thermo Scientific), following the manufacturer’s recommendations [51, 52]. Glycan antigen LAM, single lipid antigens (e.g., TDM and TMM), and multiple lipid antigen from *Mycobacterium tuberculosis* total lipids will be modified using 4-(4,6-dimethoxy [1, 3, 5] triazin-2-yl)-4-methyl-morpholinium (DMTMM) dissolved in ethanol and conjugated beads following the COOH-DMTMM method [53].

**Fig. 2:**
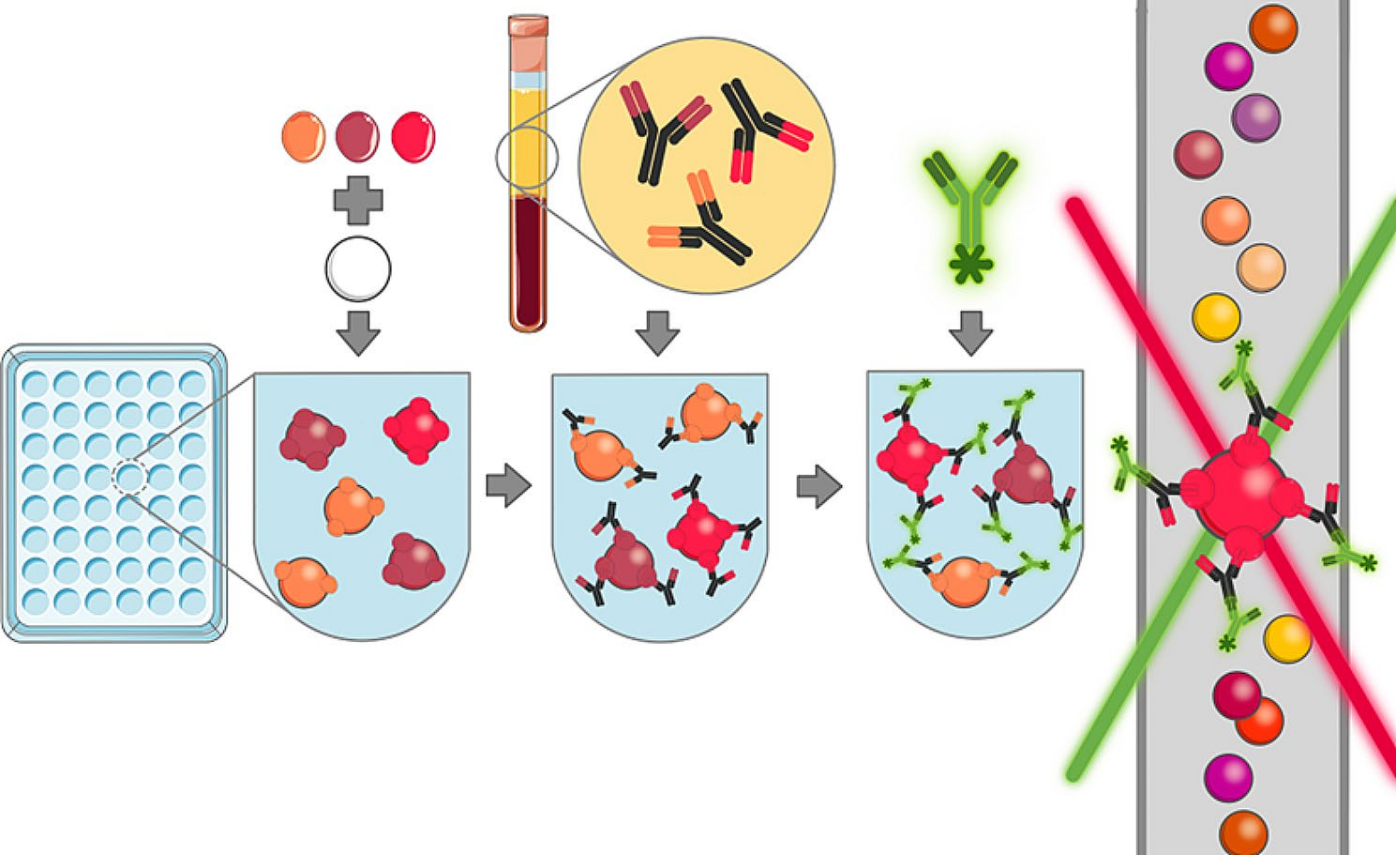
Multiplex bead-based serological assay For the multiplex bead-base serological assay (1) specific antigens are coupled to beads, (2) plasma samples are incubated with the antigen-coupled beads, allowing specific antibodies to bind to corresponding antigens, (3) fluorescently labelled detection antibodies are added, binding to antigen-specific antibodies or their properties, (4) fluorescence is measured by using a coloured laser, and concentrations are then calculated based on a standard curve

The antigen-specific antibodies concentrations and properties will be measured using different PE-labelled detection antibodies as follows: for the isotypes and subclasses, PE-coupled detection antibodies (anti-IgG, anti-IgA, anti-IgM, anti-IgG_1_, anti-IgG_2_, anti-IgG_3_ and anti-IgG_4_) at a concentration of 1 µg/mL; [52] for the FcR binding profiles, FcRs (FcγRIIIa/CD16a, FcγRIIIb/CD16b, FcγRIIa/CD32a H167, FcγRIIb/CD32b, FcγRI/CD64 from R&D Systems) will be labelled with PE and added to the samples at a concentration of 1 µg/mL; and for the glycosylation profiles, PE-labelled lectins (SNA for sialic acid, ECL for galactose, LCA for fucose and PHA-E for N-acetylglucosamine) will be used at a concentration of 20 µg/mL. After 2 h of incubation at room temperature, the beads will be washed with PBS-0.05% Tween20, and PE signal will be measured using xMAP technology. (refer to Fig. 2)

### Data management

All data will be securely entered and shared through password-protected and encrypted systems to uphold the confidentiality of health-related personal information. Adhering to Swiss legal requirements for data protection (Ordinance HRO Art. 5), our procedures for storing biological samples and handling health data are meticulously governed. Coding mechanisms and personalised logins are implemented to grant exclusive access to the study database and source documents for authorised personnel, thereby preventing third-party disclosure. Unique identification numbers are assigned to the biological samples and health-related personal data.

### Data analysis

Descriptive statistics, including mean, median, standard deviation, and interquartile range, will be used to summarise antibody concentrations stratified by diagnostic group (TB disease, TB infection, healthy TB-exposed controls, and non-TB lower respiratory tract infections) and age groups (< 2 years, 2 to < 5 years, 5 to < 10 years, and ≥ 10 years). Antigen-specific antibody concentrations will be analysed in relation to the total (nonspecific) antibody concentrations. Comparisons between groups will be made using t-tests or Mann-Whitney *U* tests if normality assumptions are not met. Children with TB disease and infection will be compared with the following groups: all other remaining children combined, healthy TB-exposed children, and children with non-TB lower respiratory tract infections.

To assess the performance of each individual antigen specific antibody feature as a diagnostic assay, sensitivity and specificity will be calculated based on cut-off values determined by the highest Youden’s index. Receiver operating characteristic (ROC) analysis will be performed, and area under the curve (AUC) will be calculated (confidence interval will be determined using the DeLong method).

In subsequent analyses, we aim to evaluate the combined interpretation of antigen-specific antibodies concentrations and properties using different strategies:

Strategy one involves defining cut-off values based on a specificity of ≥ 98%, in accordance with the minimal WHO’s target product profile(TPP) requirement for a biomarker-based detection test. We will calculate the corresponding sensitivity. Similarly, we will determine cut-off values based on a sensitivity of ≥ 66% and calculate the corresponding specificity. To assess the combined interpretation of multiple antigen targets, the test for a specific antibody or antibody property will be scored positive if at least one antibody level against a specific antigen exceeds the cut-off value in an individual’s plasma sample, and negative if all antibody levels against all antigens in a plasma sample are below the cut-off values.

Another strategy for the combined interpretation of multiple antibody concentrations and properties will involve feature selection using the least absolute shrinkage and selection operator (LASSO). This approach will help identifying the most informative features that could be used in diagnostic assays. To validate the predictive power of the selected features (k features), we will train and evaluate an additional model using only those k features. In a further step, we will include the selection of antibody concentrations and properties in the training of the model. By performing feature selection using LASSO, we aim to maximize prediction performance using all features and select the k most informative features after the training stage. This procedure is based on the concept that selecting the most informative features from a well-performing prediction model will also yield a well-performing prediction model when one only has access to the selected subset of features. Recent advances in machine learning research will enable us to incorporate feature subset selection directly into the training step of a model [54, 55]. Therefore, we optimise not only the prediction performance but also the subset selection of k features during training. The choice of subset size, k, should be based on external constraints. The diverse sensitivities and specificities observed in paediatric TB serological tests make a precise sample size determination challenging. To estimate the sample size for our experiments, we used data generated from a cohort of adults with latent infection (*n* = 20) and active pulmonary disease (*n* = 22) from South Africa [56]. For the analysis of 75 antibody features, linear regression was conducted to assess the association between diagnosis and antibody feature, while controlling for age and gender. For the thirteen features exceeding a false discovery rate threshold of 10%, the partial correlation coefficient of 0.50 or higher was observed between diagnosis and antibody feature. Using this estimate as the effect size of biologically active antibody features, 68 individuals in an independent cohort (34 LTB, 34 ATB) would provide a statistical power of 80% to observe significant differences in top antibody features between tuberculosis infection and disease at an alpha level of 0.0005. This alpha level represents the threshold for significance required by the Bonferroni-Holm correction method, set at 0.0005 to accommodate the testing of 100 antibody features.

### Publication and dissemination policy

Findings of this study will be disseminated through peer-reviewed journals, scientific conferences, and other relevant platforms. Participants will receive a summary of the results. All scientific data generated from this project will be made available as soon as possible, and no later than the time of publication or the end of the funding period, whichever comes first. The data and related metadata underlying reported findings will be deposited in a public data repository. A data access committee will support third parties who wish to perform further research with the data. Data will be curated in the repository following accepted standards and a persistent identifier, a DOI, is created for each data set published. If intellectual property is developed, dissemination of data will occur after appropriate protections for intellectual property are put in place.

## Discussion

The development of reliable point-of-care tests for detecting TB infection and disease in children is crucial. Serological assays offer a promising approach, as they may be used in a point-of-care test format, making them suitable for widespread implementation in diverse settings [7]. However, there are several hurdles that need to be addressed to advance the development of TB serological assays. One challenge is the incomplete understanding of the immunogenic properties of the numerous potential antigens of *M. tuberculosis*, including proteins and glycolipids [57]. Our study has four main strengths. First, our study will evaluate antibodies against a broad range of protein antigens [41, 45, 46, 58], as well as glycolipids that are believed to play a crucial role in the pathogenesis of *M. tuberculosis* [59, 60].

Second, to overcome the challenge of potential cross-reactivity of antibodies detected in a serological assay for TB with BCG- and non-tuberculous mycobacteria-antigens [25], we will include a large range of antibodies and reduced the overlap between *M. tuberculosis* and BCG/non-tuberculous mycobacteria-antigens selected. Third, there exists substantial interindividual heterogeneity in the antibody response to *M. tuberculosis* [61, 62]. Different individuals may react to different antigens, resulting in relatively low sensitivity but good specificity for each individual antigen serological assays [30, 31, 49]. To account for this heterogeneity, our analysis includes multiple antigen targets, such as cell wall fractions and total lipids, and aims at a combined interpretation of these parameters.

Finally, we will evaluate specific antibody properties, such as antibody isotypes, glycosylation patterns, and FcR binding profiles [12]. So far, IgG is the most extensively studied isotype and has shown the most promising results for use in diagnostic assays to detect TB disease in children. Other isotypes, such as IgA, have gained attention more recently, as these have a protective role in human and animal studies in preventing TB infection [63, 64]. Glycosylation of the Fc region affects the binding affinity of the antibody to the FcRs. Notably, distinct glycosylation patterns have been associated with various stages of TB disease and infection [11]. Lastly, our data analysis is stratified across distinct age groups to accommodate the dynamic nature of TB disease during various developmental stages of children.

The findings of our study will improve our understanding of the human humoral immune response to *M. tuberculosis* infection and disease and holds the potential to pave the way for designing antibody-based assays with high performance characteristic for use in children.

### Electronic supplementary material

Below is the link to the electronic supplementary material.

Supplementary Material 1

## Data Availability

Data supporting this study protocol is comprehensively presented within the manuscript. For additional details or inquiries regarding the dataset, kindly reach out to the Corresponding Author, Prof. Nicole Ritz, MD/PhD, nicole.ritz@unibas.ch.

## Abbreviations

AUC: Area under the curve
BCG: Bacillus Calmette-Guérin
CITRUS: Childhood tuberculosis in Switzerland study
FcR: Fragmented crystallizable region (Fc) receptor
IGRA: Interferon-γ release assay
MTB: *Mycobacterium tuberculosis*
NAAT: Nuclear acid amplification testing
LASSO: least absolute shrinkage operator
ProPAED: Procalcitonin guidance to reduce antibiotic treatment of lower respiratory tract infections in children and adolescents study
pTBred: The Spanish Pediatric Tuberculosis Research Network
ROC: Receiver operating characteristic
TB: Tuberculosis
TST: Tuberculin skin test
WHO: World Health Organization
