# Correction: Evaluation of serological assays for the diagnosis of childhood tuberculosis disease: a study protocol

**DOI:** 10.1186/s12879-024-09432-8

**Published:** 2024-06-24

**Authors:** Daniela Neudecker, Nora Fritschi, Thomas Sutter, Lenette L. Lu, Pei Lu, Marc Tebruegge, Begoña Santiago-Garcia, Nicole Ritz

**Affiliations:** 1 Mycobacterial and Migrant Health Research Group, Department of Clinical Research, University of Basel Children’s Hospital Basel, University of Basel, Spitalstrasse 33, Basel, CH 4031 Switzerland; 2 University of Basel Children’s Hospital Basel, University of Basel, Basel, Switzerland; 3 Department of Computer Science, Medical Data Science, Eidgenössische Technische Hochschule (ETH) Zurich, Zurich, Switzerland; 4 Department of Immunology, UT Southwestern Medical Center, Dallas, TX USA; 5 Parkland Health and Hospital System, Dallas, TX USA; 6 Division of Geographic Medicine and Infectious Diseases, Department of Internal Medicine, UT Southwestern Medical Center, Dallas, TX USA; 7 Department of Paediatrics, The Royal Children’s Hospital Melbourne, The University of Melbourne, Parkville, Australia; 8 Department of Infection, Immunity and Inflammation, UCL Great Ormond Street Institute of Child Health, University College London, London, UK; 9 Department of Paediatrics & National Reference Centre for Paediatric TB, Klinik Ottakring, Vienna Healthcare Group, Vienna, Austria; 10 Pediatric Infectious Diseases Department, Gregorio Marañón University Hospital, Madrid, Spain; 11 Gregorio Marañón Research Health Institute (IiSGM), Madrid, Spain; 12 Centro de Investigación Biomédica en Red de Enfermedades Infecciosas (CIBER INFEC), Instituto de Salud Carlos III, Madrid, Spain; 13 Translational Research Network in Pediatric Infectious Diseases (RITIP), Madrid, Spain; 14 Paediatric Infectious Diseases Unit, Children’s Hospital, Lucerne Cantonal Hospital, Lucerne, Switzerland

Following publication of the original article [1], we have been notified that due to a system error, the proofs were not sent to the corresponding author, meaning they were not able to submit their corrections.

The corrections that had been omitted may be seen below

Author group:

Nora Fritisch should read Nora Fritschi

Lenette Lu should read Lenette L. Lu

Table 1: “day” should read “days”

Page 6, paragraph 3: “target product profile” needs to be included before its abbreviation.

References 7, 10, 19, 39, 47 and 54 were updated for accuracy.

The original article has been corrected.
